# Gender-Related and Hemispheric Effects in Cortical Thickness-Based Hemispheric Brain Morphological Network

**DOI:** 10.1155/2020/3560259

**Published:** 2020-08-11

**Authors:** Yong-Ho Choi, Je-Yeon Yun, Bo-Hyun Kim, Min-Ho Lee, Sa-Kwang Song, Jong-Min Lee

**Affiliations:** ^1^Department of Biomedical Engineering, Hanyang University, Seoul, Republic of Korea; ^2^Seoul National University Hospital, Seoul, Republic of Korea; ^3^Yeongeon Student Support Center, Seoul National University College of Medicine, Seoul, Republic of Korea; ^4^Korea Institute of Science and Technology Information, Daejeon, Republic of Korea; ^5^University of Science and Technology, Daejeon, Republic of Korea

## Abstract

**Objective:**

The current study examined gender-related differences in hemispheric asymmetries of graph metrics, calculated from a cortical thickness-based brain structural covariance network named hemispheric morphological network.

**Methods:**

Using the T1-weighted magnetic resonance imaging scans of 285 participants (150 females, 135 males) retrieved from the Human Connectome Project (HCP), hemispheric morphological networks were constructed per participant. In these hemispheric morphologic networks, the degree of similarity between two different brain regions in terms of the distributed patterns of cortical thickness values (the Jensen–Shannon divergence) was defined as weight of network edge that connects two different brain regions. After the calculation and summation of global and local graph metrics (across the network sparsity levels *K* = 0.10‐0.36), asymmetry indexes of these graph metrics were derived.

**Results:**

Hemispheric morphological networks satisfied small-worldness and global efficiency for the network sparsity ranges of *K* = 0.10–0.36. Between-group comparisons (female versus male) of asymmetry indexes revealed opposite directionality of asymmetries (leftward versus rightward) for global metrics of normalized clustering coefficient, normalized characteristic path length, and global efficiency (all *p* < 0.05). For the local graph metrics, larger rightward asymmetries of cingulate-superior parietal gyri for nodal efficiency in male compared to female, larger leftward asymmetry of temporal pole for degree centrality in female compared to male, and opposite directionality of interhemispheric asymmetry of rectal gyrus for degree centrality between female (rightward) and male (leftward) were shown (all *p* < 0.05).

**Conclusion:**

Patterns of interhemispheric asymmetries for cingulate, superior parietal gyrus, temporal pole, and rectal gyrus are different between male and female for the similarities of the cortical thickness distribution with other brain regions. Accordingly, possible effect of gender-by-hemispheric interaction has to be considered in future studies of brain morphology and brain structural covariance networks.

## 1. Introduction

Structural MRI (sMRI) has been used in attempts to construct group brain networks by detecting whole-brain morphological connectivity patterns based on the interregional morphological similarities or “brain structural covariance” across participants (interindividual) [1] or per individual (intraindividual) [2]. On the other hand, diffusion tensor imaging (DTI) and functional magnetic resonance imaging MRI (fMRI) have been commonly used for constructing the individual brain networks connected by way of the axonal pathways (in DTI) or coupled changes of brain functional activations (in fMRI), respectively [3, 4]. The brain structural covariance network partly reflects the patterns of brain white matter-based physical connectivity [5–7], coordinated oscillations in BOLD signal changes across the whole brain during the resting status (so-called resting state functional connectivity) [8, 9], and coordinated brain development and maturation [1, 10, 11]. For the construction of brain network, the sMRI could be preferred to fMRI or DTI because of its advantages in terms of easy access, high signal-to-noise ratio, and relative insensitivity to artifacts.

The hemispheric asymmetry of the structural and functional networks has been studied because it could be an important aspect in understanding the organization of the human brain [12–14]. Interregional asymmetry studies were focused on identifying which regions and/or connections are stronger in one hemisphere than in the other. Further, representing the characteristics of the brain as a network can provide novel insights into the brain system [15]. For instance, a network of hemispheric brain asymmetry could assess the differences in the configuration (degrees of integration as well as segregation) of anatomical substrates for facilitating the information transfer between different brain regions [12]. A graph-theoretical analysis of brain networks has been applied successfully in investigating how the human brain is organized in healthy individuals and in patients with neuropsychological diseases such as Alzheimer's disease (AD), schizophrenia [16–19], major depressive disorder [20], obsessive-compulsive disorder [2], and eating disorder [21], among others. Tian et al. investigated the difference in the topologies of the hemispheric functional networks in healthy right-handed adults. The researchers showed regions that exhibited hemispheric-related differences in regions formerly observed to be functionally or structurally asymmetric [13]. Zhong et al. investigated how topological asymmetries evolve from adolescence to young adulthood and determined that rightward asymmetry in both global and local network efficiencies was consistently observed in adolescents and young adults and that the degree of asymmetry was significantly decreased in young adults [22].

Of note, gender is known as one of the key factors associated with not only the interregional asymmetry but also the hemispheric asymmetry of the brain networks. For instance, Iturria-Medina et al. studied the difference between the topological organizations of the two hemispheric structural networks in healthy right-handed individuals and discovered that the left hemisphere presented more central and essential regions, whereas the right hemisphere was more efficient and interconnected [12]. Tian et al. showed that males tend to be more locally efficient in their right hemispheric networks and females in their left hemispheric networks, which suggested that the local efficiency of the hemispheric network could be associated with behavioral and cognitive differences between men and women [13]. Caeyenberghs and Leemans found that males have a greater global efficiency of the structural hemispheric networks than do females [23].

Compared to the interindividual brain structural covariance network, intraindividual brain covariance networks could be more convenient in finding the neural correlates of specific neuropsychiatric disorders (to be used for the machine learning-based disease classification) and of specific human behaviors (by way of the calculation of correlation coefficients). Accordingly, several methods have been developed to estimate the intraindividual version of brain structural covariance network [24–26]. Tijms et al. constructed an individual network using the intensity similarity between the patches on volume space, with different numbers of nodes for each individual. Thus, it was difficult to be used for group analysis because the sizes of the constructed individual networks were generally different [24]. Kong et al. proposed to construct a morphological brain network based on interregional morphological connectivity in terms of the Kullback–Leibler (KL) divergence among a regional morphological distribution and revealed longitudinal changes in morphological connectivity of the thalamus after long-term sleep deprivation [25, 27]. However, because the KL divergence is asymmetric, the average of a corresponding pair of values was used to calculate the morphological connectivity between two regions.

To the best of our knowledge, no study has reported hemisphere-related differences in the topological organization of brain structural (morphological) covariance networks. Furthermore, very little is known about the patterns of gender-by-hemispheric interaction in terms of the global (i.e., balance of brain network integration) versus segregation and local (i.e., which brain regions well reflect the general trend of brain morphology across the brain) graph metrics. By applying the norm of Jensen–Shannon (JS) divergence for calculating the weights of network edges [28, 29], an intraindividual brain structural covariance network named “hemispheric morphological network”—that reflects the levels of similarities between different brain regions in terms of the cortical thickness distribution per participant—was constructed. We investigated (1) whether each hemispheric morphological network exhibits small-world and high efficiency properties, (2) whether the hemispheric and gender effects exist in the morphological network at the global and regional scales, and (3) whether a gender difference in the hemispheric asymmetry exists in the morphological network at the global and regional scales.

## 2. Materials and Methods

**Figure 1:**
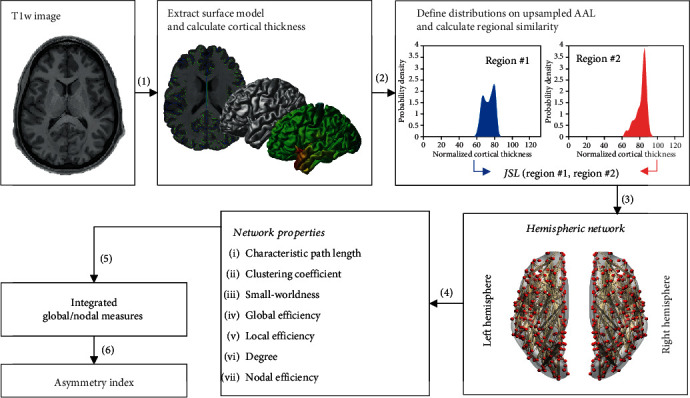
Flowchart for the construction of a hemispheric morphological network using cortical thickness. Cortical thickness was extracted from the brain surface model using CIVET pipeline v2.1 (a). The regional distributions of the cortical thickness based on the upsampled AAL atlas were estimated (b). In (c), the hemispheric morphological networks were constructed using the Jensen–Shannon similarity (JSS) based on Jensen–Shannon divergence (JSD) as the edge. The global and local graph metrics of the network were calculated and integrated (d, e). Finally, the asymmetry indexes of the integrated global and local graph metrics of the network were calculated (f).

The framework of this study design is shown in Figure 1. The details of the procedures are described below. All MRI data were analyzed using CIVET v2.1 pipeline (https://wiki.bic.mni.mcgill.ca/ServicesSoftware/CIVET), MATLAB (MATLAB R2016b, The MathWorks, Inc., Natick, Massachusetts, United States), and R [30].

### 2.1. Sample Characteristics and MRI Acquisition

The data used in this study were selected from the publicly available S900 Release of Human Connectome Project (HCP), WU-Minn Consortium [31, 32]. The samples were selected from the HCP data according to these two criteria: (1) they had a handedness score greater than or equal to 50 and (2) they did not share family members between them. A total of 285 right-handed individuals between the ages of 22 and 36 years (150 females and 135 males) were selected. The experiments were performed in accordance with relevant guidelines and regulations, and the experimental protocol was approved by the Institutional Review Board (IRB) (IRB # 201204036; Title: Mapping the Human Connectome: Structure, Function, and Heritability). Written informed consent was obtained from all participants. The data analysis was performed in accordance with the ethical guidelines of the Hanyang University Ethics Committee. The structural T1-weighted MRI was acquired on a 3T Siemens Skyra scanner using a three-dimensional magnetization prepared rapid gradient-echo (MPRAGE) sequence. The main MR parameters were the repetition time (TR) = 2.4 s, echo time (TE) = 2.14 ms, inversion time (TI) = 1000 ms, flip angle = 8, field of view (FOV) = 224 × 224 mm, and 0.7 mm isotropic voxels.

### 2.2. Cortical Surface Modeling and Extraction of Cortical Thickness Values

T1-weighted MRIs were preprocessed by PreFreeSurfer using HCP Pipelines anatomical modules [33]. And preprocessed T1-weighted MRIs were processed using CIVET v2.1 pipeline developed by the Montreal Neurological Institute (MNI). First, native MRI images were registered into a standardized stereotaxic space using an affine transformation [34]. The intensity nonuniformity resulting from the inhomogeneity in the magnetic field was corrected using N3 algorithms [35], and nonbrain tissue was excluded using the brain extraction tool (BET) algorithm [36]. Subsequently, gray matter (GM), white matter (WM), and cerebrospinal fluid were defined on the stereotaxic brain mask using an artificial neural net classifier [37]. The inner surfaces consisting of polygons of triangular components were extracted from the defined WM mask images and then expanded to the outer surfaces using the constrained Laplacian-based automated segmentation with proximities (CLASP) algorithm [38]. The cortical thickness was defined using the Euclidean distance between the linked vertices of the inner and outer surfaces, after applying an inverse transformation matrix to the cortical surfaces and reconstructing them in the native space [38, 39].

### 2.3. Construction of the Hemispheric Brain Morphological Network

The cortex was initially divided into 78 regions using the automated anatomical labeling (AAL) atlas [40] (see Table S1 in Supplementary Materials). The regions were then upsampled into 512 cortical regions with a similar number of vertices in each region [41, 42] because recent studies have highlighted the effect of region size on structural connectivity [43, 44]. We used the sphere model corresponding cerebral surface model to balance the region size in the AAL atlas. We applied *k*-means algorithm with different number of clusters to each region in order to make all the subregions of similar size. Network nodes were specified as 512 cortical regions, which contained an average of 149 vertices with a standard deviation of 22 vertices. Since we used the AAL atlas, the generated subregions maintain anatomical boundaries such as gyri and sulci. Regions within the upsampled AAL atlas are therefore approximately equal in size and maintaining anatomically constrained. Finally, 256 × 256 left- and right-hemispheric morphological networks were constructed from each subject. Network edges between two regions were defined using the Jensen–Shannon divergence (JSD), as follows:

(1) $$\begin{array}{c} \text{JSD}\left(p,q\right)=\frac{1}{2}\int p\left(x\right)\log\frac{p\left(x\right)}{\left(p\left(x\right)+q\left(x\right)\right)/2}dx+\frac{1}{2}\int q\left(x\right)\log\frac{q\left(x\right)}{\left(p\left(x\right)+q\left(x\right)\right)/2}dx. \end{array}$$

The parameters *p* and *q* are the distributions of the cortical thickness in each region, which were estimated using the kernel density estimation method [25, 45]. While the bandwidth is difficult to determine in kernel density estimation, it is associated with sample size [46, 47]. The mesh points that is sample size (*N* = 27) was experimentally determined; therefore, the bandwidth was manually determined, because the brain regions were parcellated into a similar size. Because the estimated regional distributions provide a description of brain regions, similarity based on morphological distribution can present a reasonable way of quantifying relationship between two regions. The JSD was converted to a similarity measure (JSS), as follows:

(2) $$\begin{array}{c} \text{JSS}\left(p,q\right)=e^{-\text{JSD}\left(p,q\right)}. \end{array}$$

Note that the JSS ranges from 0 to 1 for two probability distributions. Finally, each hemispheric network was binarized over sparsity ranges from 10% to 36% at 0.02 intervals. Sparsity is defined as the ratio of the total number of edges to the maximum possible number of edges in a network. The minimum sparsity calculated from the mean degree over all the nodes should be at least 2 × log(*N*), or 10%, and the maximum sparsity was chosen to allow prominent small-world properties in the brain network, at 36% [16, 48, 49]. Note that we did not covariate the constructed network matrix for any score of brain lateralization (i.e., the Edinburgh score).

### 2.4. Network Analysis: Global Graph Metrics

The global graph metrics were calculated for each hemispheric morphological network at a sparsity level of *K* = 0.10–0.36 (with 0.02 interval) using the Brain Connectivity Toolbox (BCT) [50]. The global graph metrics included the (1) normalized characteristic path length, (2) normalized clustering coefficient, (3) small-worldness, (4) global efficiency, and (5) local efficiency.

First, the characteristic path length *L*_*p*_ (a global graph metric reflecting the degree of network integration and information transfer) was derived as a harmonic mean of the shortest path lengths between two different nodes (*L*_*ij*_; *N* is the number of nodes comprising a given network (*G*), *i* ≤ *N*, *j* ≤ *N*, *i* ≠ *j*) comprising a network (*G*) [49]:

(3) $$\begin{array}{c} L_{p}={\left(\frac{\sum_{i\in G}\sum_{j\in G,j\neq i}L_{ij}^{-1}}{N\left(N-1\right)}\right)}^{-1}. \end{array}$$

Second, the clustering coefficient *C*_*p*_ (a graph metric of network segregation) was calculated by averaging the fraction of triangles around each node (or the fraction of node's neighbors that were also connected each; *k*_*i*_ is the number of nodes within a network (*G*) connected to the *i*th node, and *E*_*i*_ is the number of neighboring edges around the *i*th node) across the whole network (*G*) [49]:

(4) $$\begin{array}{c} C_{p}=\frac{1}{N}\underset{i\in G}{\sum }\frac{2*E_{i}}{k_{i}\left(k_{i}-1\right)}. \end{array}$$

In addition, the *normalized characteristic path length* and *normalized clustering coefficient* values were derived by dividing the original *L*_*p*_ or *C*_*p*_ values using the averaged values of characteristic path length or clustering coefficient for a total of 100 random networks (*L*_ran_ or *C*_ran_, respectively; constructed by random shuffling of edges within the original network, the number of nodes and edges, and the degree (number of edges connected to each node) distribution preserved) [51].

Third, the *small-worldnessσ* (a global graph metric for the balance of network integration *L*_*p*_/*L*_ran_ and network segregation *C*_*p*_/*C*_ran_) was defined as [49]

(5) $$\begin{array}{c} \sigma =\frac{\gamma }{\lambda }=\frac{C_{p}/C_{\mathrm{ran}}}{L_{p}/L_{\mathrm{ran}}}. \end{array}$$

Fourth, the *global efficiencyE*_global_ (a global graph metric of network integration and information transfer) was defined as an average of the inverse shortest path length [52]:

(6) $$\begin{array}{c} E_{\text{global}}=\frac{1}{N\left(N-1\right)}\underset{i\in G}{\sum }\underset{j\in G,j\neq i}{\sum }\frac{1}{L_{ij}}. \end{array}$$

Fifth, the *local efficiencyE*_local_ (a graph metric of local network segregation) was calculated by averaging the changed values of local clustering among the neighbors of *i*th node (*N* is the number of nodes comprising a given network (*G*), *i* ≤ *N*) when the *i*th node is deleted across the whole nodes within a network (*G*) [52]:

(7) $$\begin{array}{c} E_{\text{local}}=\frac{1}{N}\underset{i\in G}{\sum }E_{\text{local},i}. \end{array}$$

### 2.5. Network Analysis: Local Graph Metrics

The local graph metrics were calculated for each hemispheric morphological network at a sparsity level of *K* = 0.10–0.36 (with 0.02 interval) using BCT. The (1) degree centrality and (2) nodal efficiency were calculated to explore the local network properties of the hemispheric morphological network.

First, the *degree centrality* of the *i*th node *k*_*i*_ was defined as the number of links (*a*_*ij*_ is the presence (1) or absence (0) of edge connecting the *i*th and *j*th nodes within a network (*G*); *N* is the number of nodes comprising a given network (*G*), *i* ≤ *N*, *j* ≤ *N*, *i* ≠ *j*) connected to the *i*th node of a network (*G*):

(8) $$\begin{array}{c} k_{i}=\underset{j\neq i\in G}{\sum }a_{ij}. \end{array}$$

Second, the *nodal efficiency* of the *i*th node *E*_nodal,*i*_ was defined as the global efficiency value computed on the neighborhood of the *i*th node and is related to the clustering coefficient [53]:

(9) $$\begin{array}{c} E_{\text{nodal},i}=\frac{1}{N-1}\underset{j\neq i\in G}{\sum }\frac{1}{L_{ij}}. \end{array}$$

### 2.6. Network Analysis: Integrated Graph Metrics

The integrated graph metrics were obtained because a specific sparsity level selection may not be sufficient to reveal the topological properties [13]. The integrated global graph metrics were defined as follows:

(10) $$\begin{array}{c} M_{\text{global}}=\frac{\sum_{r\in R}\text{M}\text{easure}\left(r\right)}{R_{N}}. \end{array}$$

The parameter *R* is the set of sparsity values with a 0.02 interval over a range of 0.1–0.36. The parameter *R*_*N*_ is the number of elements in the set of sparsity values, and Measure(*r*) is the global network metric (*L*_*p*_, *C*_*p*_, *σ*, *E*_global_, and *E*_local_) at sparsity *r*. The integrated local graph metrics of node *i* are defined similarly as follows:

(11) $$\begin{array}{c} M_{\text{local},L_{\text{AAL}}}=\frac{\sum_{r\in R}\text{m}\text{eanMeasure}\left(L_{\text{AAL}},r\right)}{R_{N}}. \end{array}$$

The parameter *R* is the set of sparsity values with an interval 0.02 over the network sparsity range of 0.1–0.36. The parameter *R*_*N*_ is the number of elements in the set of sparsity values, and meanMeasure(*L*_AAL_, *r*) is the mean local network metric (*k*_*L*_AAL__, *E*_nodal,*L*_AAL__) of a region *L*_AAL_ at sparsity *r*. The parameter *L*_AAL_ is one of the 78 cortical regions within the AAL atlas.

### 2.7. Asymmetry Index of Global and Local Graph Metrics

For the global and local graph metrics, the asymmetry indexes (AIs) of the hemispheric morphological network were calculated as follows:

(12) $$\begin{array}{c} \text{AI}\left(M\right)=100*\frac{2*\left(M_{L}-M_{R}\right)}{\left(M_{L}+M_{R}\right)}. \end{array}$$

The parameters *M*_*R*_ and *M*_*L*_ are the integrated global and local graph metrics of the right and left hemispheric networks, respectively. The integrated global and local graph metrics of the hemispheric network were used as the summary of the graph metrics over the sparsity range. Note that positive AI(*M*) means leftward asymmetry and negative AI(*M*) rightward asymmetry.

### 2.8. Statistical Analyses

A two-way repeated-measures analysis of variance (ANOVA) was performed with hemisphere (left and right) as a repeated-measures factor, gender as a between-subject factor, and age as covariate, to determine whether there were significant effects on any of the global graph metrics. A one-sample *t*-test within each gender was performed to determine whether the global and local metrics in the hemispheric networks of each gender showed asymmetry. A two-sample *t*-test between each gender was performed to identify whether the AIs of the global and local metrics were different. The significance threshold of the statistical analysis was set at *p* < 0.05 for the global and local integrated metrics. The significance threshold was set at the false discovery rate- (FDR-) corrected value *p* < 0.05 for the local graph metrics. All statistical analyses were performed in R.

## 3. Results

### 3.1. Small-Worldness and Global/Local Efficiencies of Hemispheric Brain Morphological Networks

**Figure 2:**
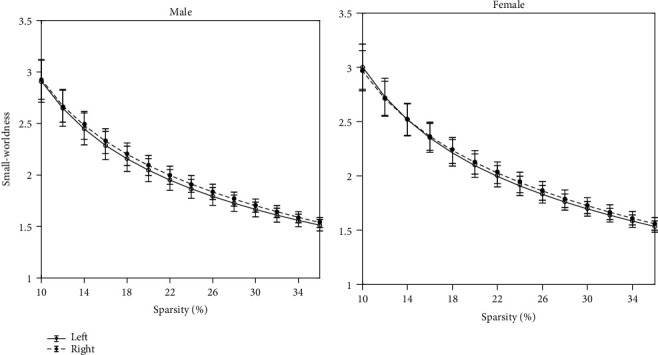
The averaged small-worldness of the hemispheric morphological network. The small-worldness was averaged across the individuals within the group of females and the group of males, respectively. The averaged small-worldness was plotted over the sparsity range of *K* = 0.1–0.36. For both male and female, the hemispheric brain morphological networks showed small-worldness since averaged small-worldness is larger than 1.5 over the entire range.

**Figure 3:**
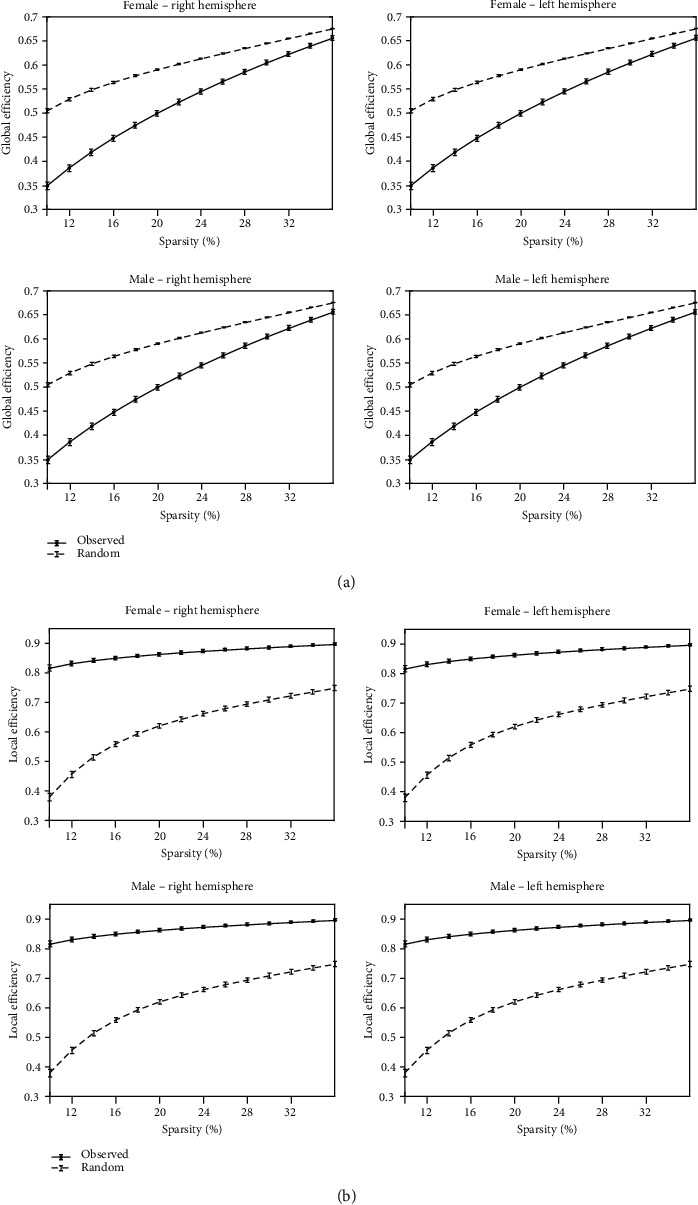
The averaged (a) global efficiency and (b) local efficiency of the hemispheric morphological network. The global and local efficiencies were averaged across the individuals within the group of females and the group of males, respectively. The global and local efficiencies were plotted over the sparsity range of *K* = 0.1–0.36. For both male and female, the hemispheric brain morphological networks showed higher global and local efficiencies than the matched random networks over the entire range. Note that “Observed” is hemispheric morphological network and “Random” is matched random network. The findings suggest that information processing within each hemisphere could be of similar efficiency to that of the whole brain.

For both male and female, the hemispheric brain morphological networks showed small-worldness since *σ* is larger than 1.5 over the entire range (see Figure 2) [49], in addition to the higher *E*_local_ and lower *E*_global_ than those of the matched random networks (see Figure 3) in the network sparsity range of *K* = 0.10–0.36. The results are consistent with those of previous hemispheric brain network studies [13, 14].

### 3.2. Gender and Hemispheric Effect on Global and Local Graph Metrics

**Figure 4:**
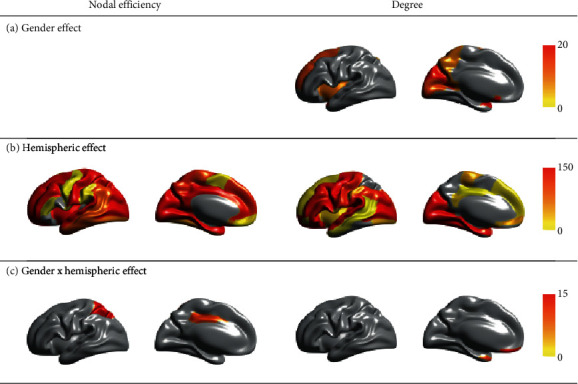
Significant (a) gender, (b) hemisphere, and (c) interaction effects on the regional integrated measures of the hemispheric network. Two-way repeated-measures ANOVA was performed to investigate various effects on the regional integrated measures. The significance level was set as FDR-corrected value *p* < 0.05. The color bar represents the *F* values.

The asymmetries of global graph metrics showed a significant hemisphere effect on *σ* (*F*_*σ*_ = 10.227, *p*_*σ*_ < 0.05), a significant gender effect on *C*_*p*_ (*F*_*Cp*_ = 4.062, *p*_*Cp*_ < 0.05), and hemisphere-gender interaction effects on *L*_*p*_ and *E*_global_ (*F*_*Lp*_ = 6.595, *p*_*Lp*_ < 0.05; *F*_*E*global_ = 4.846, *p*_*E*global_ < 0.05) (see Table S2). The asymmetries of local graph metrics showed significant hemisphere effects (FDR-corrected *p* < 0.05) on 38 nodes and significant hemisphere-gender interaction effects (FDR-corrected *p* < 0.05) on 2 nodes (see Figure 4). For the degree, we observed a significant hemisphere effect (FDR-corrected *p* < 0.05) on 33 nodes, a significant gender effect (FDR-corrected *p* < 0.05) on 7 nodes, and a significant hemisphere-gender interaction effect (FDR-corrected *p* < 0.05) on 2 nodes (see Figure 4). Significant hemispheric effects on two local graph metrics were observed on nearly the same nodes. Both global and local characteristics of morphological network showed not only hemispheric effect but also gender effect. Furthermore, there is interaction effect also in the global and local characteristics of morphological network.

### 3.3. The Asymmetry Index of the Global and Local Graph Metrics within Groups

Females showed significant rightward hemispheric asymmetries in *L*_*p*_ (*t*_*Lp*_ = –3.551, *p*_*Lp*_ < 0.01) and significant leftward hemispheric asymmetry in *E*_global_ (*t*_*E*glob_ = 2.873 and *p*_*E*glob_ = 0.05). Males showed significant rightward hemispheric asymmetry in *σ* (*t*_*σ*_ = –3.662, *p*_*σ*_ < 0.01) (Table 1).

**Figure 5:**
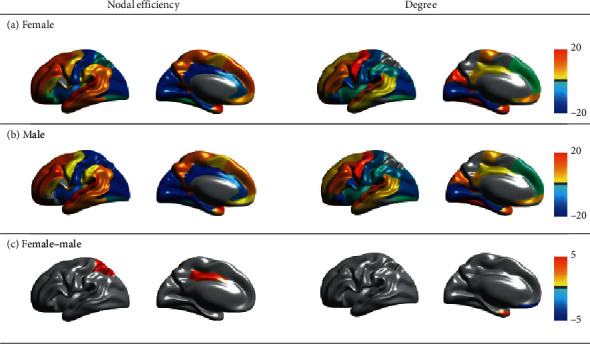
Significant asymmetry related to gender on the regional integrated measures of the hemispheric network. In (a) and (b), one-sample *t*-tests were performed to investigate the asymmetries of the regional integrated measures within gender. The significance level was set as the FDR-corrected value *p* < 0.05. The color bar represents the *t* values. Red–yellow indicates leftward asymmetry, and blue–cyan indicates rightward asymmetry. In (c), two-sample *t*-test was performed to investigate the gender differences on the asymmetries of the regional integrated measures between the genders. The significance level was set as the FDR-corrected value *p* < 0.05. The color represents the *t* values. A larger positive or negative *t* value indicates a greater leftward or rightward asymmetry in one group than in the other.

For asymmetries of nodal global efficiency, females showed significant asymmetries on 35 nodes (20 leftward asymmetries and 15 rightward asymmetries), and males showed significant asymmetries on 29 nodes (15 leftward asymmetries and 14 rightward asymmetries) at the FDR-corrected *p* value < 0.05 (Figure 5); asymmetries of nodal global efficiencies are in the same direction on 28 nodes in both females and males. For asymmetries of degree centrality, females showed significant asymmetries on 28 nodes (12 leftward asymmetries and 16 rightward asymmetries), and males showed significant asymmetries on 31 nodes (16 leftward asymmetries and 15 rightward asymmetries) at the FDR-corrected *p* value < 0.05 (see Figure 5). The asymmetries of the females and males are in the same direction in 26 nodes. While local graph metrics showed leftward and rightward asymmetries together in females and males, global graph metrics of the morphological network tended leftward in both females and males.

### 3.4. Between-Group Differences in Asymmetry Index of Global and Local Graph Metrics

For the asymmetries of global graph metrics, significant group differences were observed on *C*_*p*_, *L*_*p*_, and *E*_global_ (*t*_*Cp*_ = –2.040, *p*_*Cp*_ = 0.042, AI_Female,*Cp*_ < AI_Male,*Cp*_; *t*_*Lp*_ = –2.846, *p*_*Lp*_ = 0.005, AI_Female,*Lp*_ < AI_Male,*Lp*_; *t*_*E*global_ = 2.479, *p*_*E*global_ = 0.014, and AI_Female,*E*global_ > AI_Male,*E*global_) (see Table 1). The females showed significantly greater rightward asymmetry in *L*_*p*_ and significantly greater leftward asymmetry in *E*_global_. Note that the degree of asymmetry in the females is greater. While the females showed rightward asymmetry, the males showed leftward asymmetry on *C*_*p*_.

For asymmetries of nodal global efficiency, the males showed greater rightward asymmetries on 2 nodes (median cingulate and paracingulate gyri, *t*_*E*nodal,MCG_ = 3.942, FDR-corrected *p*_*E*nodal,MCG_ < 0.05, mean of AI_Female,*E*nodal,MCG_ = –1.771, mean of AI_Male,*E*nodal,MCG_ = –2.285; superior parietal gyrus, *t*_*E*nodal,SPG_ = 4.532, FDR-corrected *p*_*E*nodal,SPG_ < 0.05, mean of AI_Female,*E*nodal,SPG_ = –0.525, mean of AI_Male,*E*nodal,SPG_ = –1.039) (see Figure 5). For asymmetries of degree centrality, the males showed leftward asymmetries, and the females showed rightward asymmetries on 1 node (the gyrus rectus, *t*_Degree,REC_ = –5.469, FDR-corrected *p*_Degree,REC_ < 0.05, mean of AI_Female,Degree,REC_ = –3.536, mean of AI_Male,Degree,REC_ = 3.997) and showed greater leftward asymmetries on 1 node (temporal pole: middle temporal gyrus, *t*_Degree,TPOmid_ = 3.395, FDR-corrected *p*_Degree,TPOmid_ < 0.05, mean of AI_Female,Degree,TPOmid_ = 30.141, mean of AI_Male,Degree,TPOmid_ = 20.892).

Opposite directionality of asymmetries (leftward versus rightward) was shown for global metrics of clustering coefficient, characteristic path length, and global efficiency. For the local metrics, larger rightward asymmetries of cingulate-superior parietal gyri for nodal global efficiency in male compared to female, larger leftward asymmetry of temporal pole for degree centrality in female compared to male, and opposite directionality of interhemispheric asymmetry of rectal gyrus for degree centrality between female (rightward) and male (leftward) were shown.

## 4. Discussion

The current study examined gender-related differences in hemispheric asymmetries of graph metrics, calculated from cortical thickness-based brain structural covariance network named hemispheric morphological network. Hemispheric morphological networks satisfied small-worldness and global efficiency for the network sparsity ranges of *K* = 0.10–0.36. Between-group comparisons (female versus male) of asymmetry indexes revealed opposite directionality of asymmetries (leftward versus rightward) for global metrics of clustering coefficient, characteristic path length, and global efficiency. For the local metrics, larger rightward asymmetries of cingulate-superior parietal gyri for nodal global efficiency in male compared to female, larger leftward asymmetry of temporal pole for degree centrality in female compared to male, and opposite directionality of interhemispheric asymmetry of rectal gyrus for degree centrality between female (rightward) and male (leftward) were shown. The overall results of this study indicate that brain network analysis using morphological features provides insights into the understanding of hemispheric asymmetry related to gender.

### 4.1. Economical Small-World Network Properties of the Hemispheric Brain Morphological Networks

The identification of economical small-world properties of the human brain network could help in the understanding of the human brain [4, 15]. It has been shown that the whole brain network of healthy subjects is characterized by economical small-world properties [3, 54–56]. In an individual morphological brain network, the presence of economical small-world properties has been reported as convergent evidence [24, 25, 57]. Furthermore, the presence of economical small-world network properties in hemispheric networks was reported using sMRI and fMRI [12, 13, 58]. Our results extend previous findings by showing that individual hemispheric morphological networks also demonstrate economical small-world properties, indicating that information processing within each hemisphere could be of similar efficiency to that of the whole brain.

### 4.2. Gender-Related Effects in Asymmetry of Global Graph Metrics

The left hemisphere in females may be more efficient in the exchange of information in parallel. The *E*_global_ in females shows leftward asymmetry. Although the left and right hemispheres of the human brain consistently communicate with each other, asymmetry of the human brain hemispheric network and its differences with respect to gender have been reported. For instance, Iturria-Medina et al. showed that the right hemisphere is more efficient with rightward *E*_global_ and *E*_local_ than the left hemisphere in right-handed individuals [12]. Shang et al. showed that the functional left hemispheric network is more globally efficient than the functional right hemispheric network, and Sun et al. reported, using the structural hemispheric network, that the left hemispheric network is globally efficient [59, 60]. Gong et al. showed that the cortical anatomical network is more efficient globally and locally in females than in males [61]. Tian et al. reported that the two hemispheres and genders are not significantly different in transferring information between the brain regions; however, females and males are both more globally efficient in their right hemispheres [13]. These findings are different, depending on the modality and methodology used. Some aspects of asymmetry observed in this study are reflected observations based on the structural and functional networks. This study suggests that the left hemispheric morphological network is more globally efficient in females, in accordance with previous observations based on the structural and functional networks [59–61].

### 4.3. Gender-Related Effects in Asymmetry of Local Graph Metrics

It was observed that males and females have similar asymmetric patterns in nodal global efficiency and degree centrality. Significant leftward asymmetries were observed in the frontal region, precuneus, and temporal region, while significant rightward asymmetries were observed in the cingulate gyrus and occipital gyrus. These patterns have been observed to be structurally asymmetric in previous studies [62]. This asymmetry pattern along the fronto-occipital axis is similar to that reported by Plessen et al. [63] and may be related to the Yakovlevian torque, where frontal/occipital bending in the human brain is present [64]. However, some regions related to behavior (properties) differences showed gender difference in asymmetry. A gender difference in asymmetries was observed in four brain regions (*E*_nodal_: the MCG, SPG; Degree: REC and TPOmid) in this study (see Figure 5). Most of these regions, such as the MCG, SPG, and TPOmid, have been reported as hubs in previous studies based on the structural and functional networks [65]. Given that the left hemisphere is dominant in language processing and the right hemisphere is dominant in spatial processing [66], the asymmetries of the local graph metrics may underlie advantageous verbal processing in females and advantageous spatial processing in males [67]. The nodal global efficiency in the MCG and SPG regions shows more rightward asymmetry in males than in females. In particular, the visuospatial processing regions, such as the right SPG, showed greater nodal global efficiency in males, which may directly contribute to the previous observation [68, 69]. These regions were also reported in the studies on the nodal global efficiency of the brain network [22, 23, 61]. For instance, Zhong et al. reported that the nodal global efficiency of the hemispheric structural network is more rightward asymmetric on the MCG and SPG. Caeyenberghs and Leemans observed rightward asymmetry of the nodal global efficiency in the MCG region using the structural network [23]. The rightward asymmetry of the nodal global efficiency in these regions is consistent with those found in previous studies using the structural network. The right hemispheric morphological network is more locally efficient in males than in females. The degree centrality in TPOmid region shows more leftward asymmetry in females than in males. The structural leftward asymmetry of the TPOmid region was studied [70, 71]. Price reported left hemispheric dominance for language in the middle temporal pole [72]. The significant leftward asymmetry of the degree centrality in the TPOmid regions is consistent with those found in previous studies. The degree centrality in the REC region shows rightward asymmetry in females and leftward asymmetry in males. The REC region is known as a nonfunctional gyrus [73]; hence, it is difficult to interpret gender difference from this region. For example, Belfi et al. reported that the REC region, a narrow strip of the cerebral cortex, has a larger volume in females; however, the gender difference could be in terms of psychological gender rather than biological gender. Therefore, higher femininity scores in gender assessments of males were associated with a larger volume of the REC region [74]. The opposing directions of asymmetry of the degree centrality in the REC regions between females and males might reflect the individual's personality rather than the biological gender.

### 4.4. Limitations

There are still some limitations in this study. First, the selection of the brain atlas could affect the topological properties of the individual brain network [57]. In the future, it is important to validate this experiment with a different atlas. Second, we used cortical thickness as a morphological feature to construct a morphological brain network. Several recent studies have reported methodologies to construct networks using multiple features [75, 76]. In the future, it would be interesting to use multiple features to construct the brain network using our method. However, it is noticeable that an exact physiological explanation of this network is still difficult. Third, we observed significant hemisphere and gender effects in topological properties of the morphological brain network. Some aspects of these effects observed in this study are consistent with those based on structural and functional brain networks, while others are not [13, 61]. Because morphological similarity is related to the underlying axonal connectivities [77], a further study simultaneously evaluating the topologies of structural networks and of functional networks is expected [78–80]. Fourth, we observed significant asymmetries related to gender in topological properties of the morphological network. Brain asymmetry is closely related to lateralized behaviors. Thus, further analysis should be implemented for a better understanding of the basis of lateralized functions such as language and visuospatial processing. Finally, changes in brain asymmetries are closely related to the pathophysiology of various brain diseases such as schizophrenia and Alzheimer's disease. Therefore, evaluating the topological organization of morphological brain networks within hemispheres is likely to improve our knowledge about the pathology of various brain diseases.

## 5. Conclusions

In this study, hemispheric morphological brain networks were constructed, and graph-theoretical approaches were used to examine the hemispheric asymmetry in females and males. First, the hemispheric morphological networks showed small-world properties and a high efficiency, similar to those of structural and functional networks. Second, the hemispheric asymmetry within and between the gender groups was investigated. In the global scale, males tend to be locally efficient in the left hemispheric network, and females are more globally efficient in the left hemispheric network. In the local scale, the right asymmetry in the nodal global efficiency is greater around the cingulate gyrus and superior parietal gyrus in males than in females. The left asymmetry in degree centrality is greater around the temporal pole in females than in males. These findings may provide evidence for the topological difference in the hemispheric morphological network and for the behavioral differences related to gender. The overall results of this study indicate that brain network analysis using morphological features provides insights into the understanding of hemispheric asymmetry related to gender.

## Figures and Tables

**Table 1:** Asymmetry of the global integrated measures of the hemispheric network within/between the gender groups. A one-sample *t*-test was performed to investigate the asymmetries of the global integrated measures within gender. The positive *t* values in females and males indicate leftward asymmetry, and the negative *t* values in females and males indicate rightward asymmetry. A two-sample *t*-test was performed to investigate the gender difference in the asymmetries of the global integrated measures between genders. The greater positive and negative *t* values indicate greater leftward or rightward asymmetry in one group than in the other. The significance level for all analyses is *p* < 0.05. *C*_*p*_,  *L*_*p*_, *σE*_global_, and *E*_local_ denote the clustering coefficient, characteristic path length, small-worldness, global efficiency, and local efficiency, respectively. The significance (*p* < 0.05) is indicated by the bold text and indicator (^∗^).

		*C*_*p*_	*L*_*p*_	*σ*	*E*_global_	*E*_local_
Within females (left–right)	*t* value	-1.381	**-3.551** ^∗^	-1.947	**2.873** ^∗^	-0.861
	(*p* value)	(0.168)	(0.000)	(0.054)	(0.005)	(0.395)
Within males (left–right)	*t* value	1.522	0.576	**-3.662** ^∗^	-0.689	1.481
	(*p* value)	(0.131)	(0.565)	(0.000)	(0.486)	(0.143)
Between groups (females–males)	*t* value	**-2.009** ^∗^	**-2.836** ^∗^	1.489	**2.479** ^∗^	-1.637
	(*p* value)	(0.042)	(0.006)	(0.152)	(0.015)	(0.100)

## Data Availability

The brain MRI dataset analyzed during this study is available in the Human Connectome Project repository (http://www.humanconnectome.org/).

## Supplementary Materials

Supplementary materials Table S1: abbreviations for the brain regions of interest. Table S2: hemisphere and gender effects on the global integrated measures of the hemispheric network revealed by two-way repeated-measures ANOVA. Two-way repeated-measures ANOVA was performed to investigate hemisphere and gender effects and interaction effects on the global integrated measures. The significance level for all analyses is *p* < 0.05. *C*_*p*_, *L*_*p*_, *σ*, *E*_global_, and *E*_local_ denote the clustering coefficient, characteristic path length, small-worldness, global efficiency, and local efficiency, respectively. The significance (*p* < 0.05) is indicated by the bold text and indicator (∗).
