# Supplementary material for: Gender-Related and Hemispheric Effects in Cortical Thickness-Based Hemispheric Brain Morphological Network
Source: Biomed Res Int. 2020 Aug 11;2020:3560259. doi: 10.1155/2020/3560259 (PMC7439209; doi:10.1155/2020/3560259)
Supplement: Supplementary materials — Table S1: abbreviations for the brain regions of interest. Table S2: hemisphere and gender effects on the global integrated measures of the hemispheric network revealed by two-way repeated-measures ANOVA. Two-way repeated-measures ANOVA was performed to investigate hemisphere and gender effects and interaction effects on the global integrated measures. The significance level for all analyses is p < 0.05. Cp, Lp, σ, Eglobal, and Elocal denote the clustering coefficient, characteristic path length, small-worldness, global efficiency, and local efficiency, respectively. The significance (p < 0.05) is indicated by the bold text and indicator (∗). [file 3560259.f1.docx]

## Supplementary Material

Table S1. Abbreviations for the brain regions of interest

| **Abbreviation** | **Region Name** | **Abbreviation** | **Region Name** |
| --- | --- | --- | --- |
| PreCG | Precentral gyrus | CAL | Calcarine fissure and surrounding cortex |
| SFGdor | Superior frontal gyrus, dorsolateral | CUN | Cuneus |
| ORBsup | Superior frontal gyrus, orbital part | LING | Lingual gyrus |
| MFG | Middle frontal gyrus | SOG | Superior occipital gyrus |
| ORBmid | Middle frontal gyrus orbital part | MOG | Middle occipital gyrus |
| IFGoperc | Inferior frontal gyrus, opercular part | IOG | Inferior occipital gyrus |
| IFGtriang | Inferior frontal gyrus, triangular part | FFG | Fusiform gyrus |
| ORBinf | Inferior frontal gyrus, orbital part | PoCG | Postcentral gyrus |
| ROL | Rolandic operculum | SPG | Superior parietal gyrus |
| SMA | Supplementary motor area | IPL | Inferior parietal |
| OLF | Olfactory cortex | SMG | Supramarginal gyrus |
| SFGmed | Superior frontal gyrus, medial | ANG | Angular gyrus |
| ORBsupmed | Superior frontal gyrus, medial orbital | PCUN | Precuneus |
| REC | Rectus gyrus | PCL | Paracentral lobule |
| INS | Insula | HES | Heschl gyrus |
| ACG | Anterior cingulate and paracingulate gyri | STG | Superior temporal gyrus |
| MCG | Median cingulate and paracingulate gyri | TPOsup | Temporal pole: superior temporal gyrus |
| PCG | Posterior cingulate gyrus | MTG | Middle temporal gyrus |
| PHG | Parahippocampal gyrus | TPOmid | Temporal pole: middle temporal gyrus |
|  |  | ITG | Interior temporal gyrus |

Table S2. Hemisphere and gender effects on the global integrated measures of the hemispheric network revealed by two-way repeated-measures ANOVA. Two-way repeated-measures ANOVA was performed to investigate hemisphere and gender effects, and interaction effects on the global integrated measures. The significance level for all analyses is p < 0.05. $\boldsymbol{C}_{\boldsymbol{p}}\boldsymbol{,}\boldsymbol{L}_{\boldsymbol{p}}\boldsymbol{, \sigma,}\boldsymbol{E}_{\boldsymbol{global}}\boldsymbol{,}{and\boldsymbol{E}}_{\boldsymbol{local}}$denote the clustering coefficient, characteristic path length, small-worldness, global efficiency, and local efficiency, respectively. The significance (p < 0.05) is indicated by the bold text and indicator, **^*^**.

|  | | $\boldsymbol{C}_{\boldsymbol{p}}$ | $\boldsymbol{L}_{\boldsymbol{p}}$ | $\boldsymbol{\sigma}$ | $\boldsymbol{E}_{\boldsymbol{global}}$ | $\boldsymbol{E}_{\boldsymbol{local}}$ |
| --- | --- | --- | --- | --- | --- | --- |
| Hemisphere effect | F-value  (p-value) | 0.003  (0.957) | 3.724  (0.054) | **10.227^*^**  (0.001) | 2.098  (0.149) | 0.090  (0.764) |
| Gender effect | F-value  (p-value) | **4.062^*^**  (0.044) | 0.001  (0.981) | 1.334  (0.249) | 0.311  (0.578) | 2.987  (0.085) |
| Interaction effect | F-value  (p-value) | 2.610  (0.107) | **6.595^*^**  (0.011) | 1.240  (0.266) | **4.846^*^**  (0.028) | 1.810  (0.179) |
